# Increased urinary volumes in symptomatic Ménière’s Disease

**DOI:** 10.1186/s13104-019-4870-3

**Published:** 2019-12-26

**Authors:** Federica Di Berardino, Diego Zanetti

**Affiliations:** 1 Audiology Unit, Dept of Clinical Sciences and Community Health, Fondazione IRCCS Ca’ Granda Ospedale Maggiore Policlinico, University of Milan, Via Pace 9, 20122 Milan, Italy; 2 Dept. of Specialistic Surgical Sciences, Fondazione IRCCS Cà Granda, Ospedale Maggiore Policlinico, Milan, Italy

**Keywords:** Urine, Volume, Mannitol, Meniere, Altered intestinal permeability, Double sugar test

## Abstract

**Objective:**

The purpose of the research is to test the measurement of the total urinary volume, induced by the diuretic osmotic action of mannitol, in a group of symptomatic MD patients and in healthy controls.

**Results:**

An altered excretory urinary volume after mannitol challenge was observed in symptomatic MD (874.3 ± 302.1) compared to healthy volunteers (361.7 ± 181.6) (p = 0.0001). This easy and self-administered method might be proposed to replace the analysis of the urinary sugars’ concentration in symptomatic MD patients.

## Introduction

In 1923, inhalant and food allergies have been related to Ménières’ disease (MD) [1]; from then until now, this relation has been reported in the literature [2–7]. Since 2013, increased intestinal permeability has been shown to be an intrinsic trait in a subset of food allergic subjects [8, 9].

The urinary excretion of two orally administered non-metabolizable sugars, lactulose and mannitol, also known as the “double sugar test” is a well-known, validated, non-invasive method to evaluate the intestinal permeability, because their urinary concentration is an indirect index of their intestinal absorption [10]. The results are expressed as the ratio of lactulose and mannitol recovered in the urine; it is a reliable marker, independent from the etiology of altered intestinal permeability and from the methods of collection [11]. It has been also considered for screening purposes in different clinical conditions, especially in children, in order to avoid more invasive tests, or in the assessment of the responses to new treatments [12].

By means of this method, we recently identified an altered intestinal permeability in symptomatic Ménière disease (MD) patients [13]. In particular, the most consistent rise was observed for mannitol (vs. lactulose) in symptomatic MD patients; therefore, we hypothesized the possible use of the mannitol challenge as a stand-alone test to detect a condition of altered intestinal permeability. We also noticed that an increase of urinary mannitol was also associated with an increase in the total volume of the urines, but this finding was initially considered less relevant than the ratio between the concentration of the two sugars. Mannitol infusion induces polyuria by an osmotic mechanism: the total diuresis exceeds 3000 ml/24 h and the osmolarity reaches 300 mOsmol/L [14].

The aim of this study was to check if the measurement of the total urinary volume, induced by the diuretic osmotic action of mannitol, can replace the analysis of the urinary sugars’ concentration. For this reason, we compared the total urine volume collected in the 4 h following the water load containing a pre-determined mannitol amount in symptomatic MD patients and in a healthy control group.

## Main text

### Methods

#### Patients

The study included 19 adult subjects extracted from a pool of 186 unilateral definite MD patients recruited at the outpatient clinic of the Vestibular Disorders Unit in a tertiary referral university Hospital during the last 3 years. They were 12 females and 7 males (mean age: 57.0 ± 10.8 years). They fulfilled all criteria for definite MD according to the AAO-HNS guidelines [15] and were negative for retrocochlear lesions at magnetic resonance imaging.

MD patients were included if:

1. altered intestinal permeability was previously confirmed by the validated “double sugar test” and faecal calprotectin > 50 μg/g;
2. they were “symptomatic” from a vestibular point of view, i.e. they suffered at least two major episodes of vertigo per month with sensorineural hearing loss and aural fullness during the 3 months prior to the admission, and
3. had a functional level (FL) ≥ 4, according to the consensus paper guidelines [15].

Exclusion criteria were:

1. a positive history for gastrointestinal disease, hypertension, celiac or bowel disease;
2. abnormal thyroid hormones;
3. history of malignant tumours or autoimmune diseases;
4. specific diets (including low-salt [Na/K] intake).

Those patients who had previously received systemic steroids or intratympanic injections (either gentamicin or steroids) were also excluded from the study, as well as those being treated with diuretics, protonic pump inhibitors and/or antihistamines.

#### Controls

Fourteen healthy volunteers (10 females, 4 males, mean age: 42.0 ± 17.0 years) served as a “control group”, in order to check the reference values. All healthy subjects were normally hearing, reported a negative familiar and personal history of vertigo or dizziness, and never suffered from otological diseases. In the inclusion criteria, they should have never suffered from gastrointestinal diseases, they should have a negative familiar history for intestinal bowel disease or celiac disease. They had a negative “double sugar test” and faecal calprotectin was < 50 μg/g.

#### Test

The standardized procedure described as the “double sugar test” [13] was modified by testing only mannitol. When awakening in the morning, each patient was instructed to collect and evaluate the volume of a pre-test urine sample. Then, they were asked to drink a solution containing 1 g mannitol dissolved in 200 ml water. Urines were collected during the next 4 h and the total volume was measured. Patients were instructed to avoid eating (not even a chewing-gum), drinking or smoking during the test, but were allowed to drink a fixed dose of water (200 ml), only half an hour after the test start.

#### Statistical analysis

Statistical analysis was performed using the SPSS statistical package version 24.00 (SPSS Inc., Chicago, Illinois). The significance of difference between the two groups compared with each other was evaluated by U-Mann–Whitney test for independent samples. Results are expressed as means and standard deviations (SD). A p < 0.05 was considered statistically significant.

### Results

MD patients were homogeneous as per onset time of MD (> 5 years), degree of hearing loss (stage 2) and absence of comorbidities. MD and control group resulted comparable for age and sex.

The volumes of urine collected upon awakening (baseline) were similar in the two groups: 323.7 ± 138.4 (MD) vs 393.2 ± 299.5 (controls), (p = n.s).

**Fig. 1:**
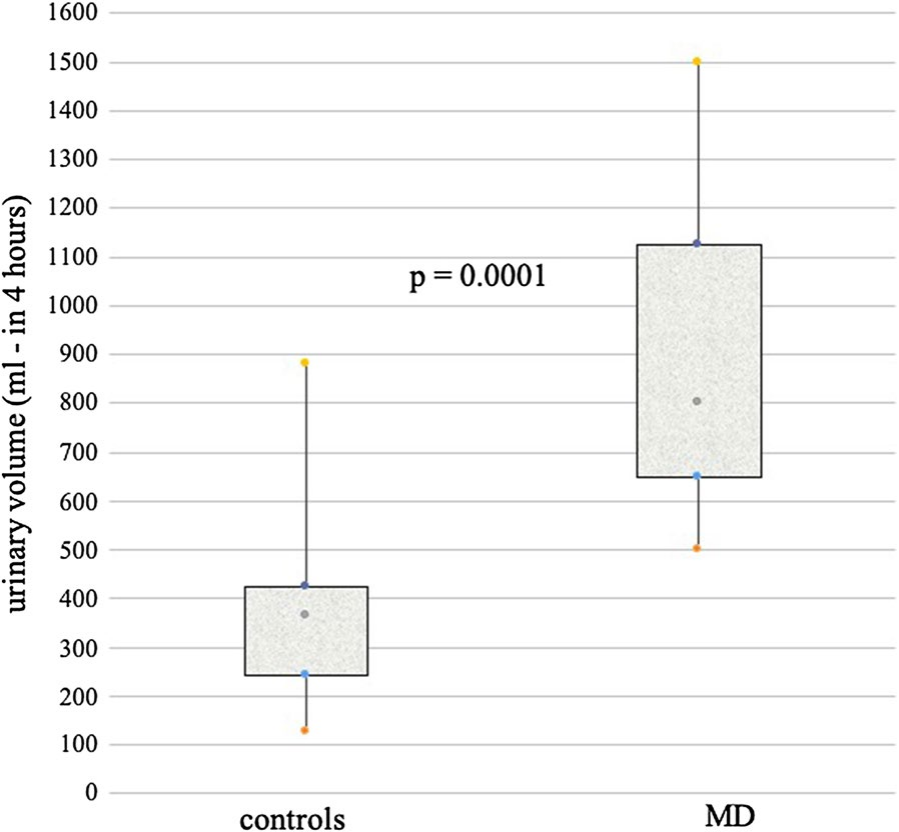
Urinary volumes collected for four hours following the water load containing mannitol in symptomatic MD and healthy controls

A significant increase of volume of the urine collected for 4 h following the water load containing mannitol was found in symptomatic MD, compared to healthy volunteers: 874.3 ± 302.1 vs 361.7 ± 181.6 (p = 0.0001), respectively (Fig. 1).

### Discussion

Mannitol is an osmotic diuretic used to prevent acute tubular necrosis [16]. It is occasionally useful in enhancing the diuresis in patients with severe resistant oedema [17]. It has been administered also to MD patients with the purpose of possibly reducing the endolymphatic hydrops [18, 19].

In healthy subjects, about 14% of mannitol administered orally is absorbed through the hydrophilic pores of the enterocytes. In the active phases of MD, we previously observed an increase of mannitol and lactulose intake as expression of the altered intestinal permeability [13]. We then speculated that the mucosal inflammation related with food or aeroallergens intolerance, unleashed by immunological cross-reaction in symptomatic MD patients, could have impaired the intestinal barrier function and induced a greater sugar absorption. Mannitol was the most sensitive between the two sugar challenges in this respect.

Mannitol behaves like an osmotic diuretic, i.e. it increases the excretion of water by the kidneys and the global volume of urine. Its mechanism of action does not involve a specific site in the kidney: it accumulates in the intercellular space, drawing water out of the cells due to increased local osmolarity. The fluids accumulated in the interstitial spaces are then rapidly eliminated as urine. An increased absorption of mannitol in case of altered intestinal permeability determines a decrease in sodium and an increase in serum osmolarity a consequently an increase in diuresis [12].

The peculiar physical and chemical properties of mannitol make it an ideal tool to test the osmotic fluid retention in different condition of intestinal absorption, given the reliability of the “*single paracellular permeation model*”, regardless of the cause of the altered intestinal permeability [20].

Our new preliminary observations in a selected sample of MD patients in an active stage of the disease (1) confirm the hypothesis of an altered intestinal permeability on the basis of a single sugar test (instead of the double-sugar); (2) may suggest the assessment of intestinal permeability by a mannitol challenge without dosage of its urinary concentration but simply measuring the diuresis of the 4 h following the water + mannitol load.

This proposed method would significantly reduce costs, compared to the validated non-invasive “double-sugar test”, providing an easy and self-administered way to identify altered intestinal permeability.

As far as we know, this is the first report of increased excretory urinary volume after mannitol challenge in a group of symptomatic MD patients.

The strength of this work is the very selected and homogeneous sample population, that includes only symptomatic, untreated MD patients, with no drug interferences.

## Limitations

The main drawback is the small size of the sample group and, in order to validate this method, further studies should be performed on larger populations. The method should be tested also in other diseases with altered intestinal permeability conditions, in order to define if this finding is peculiar of MD. Other serologic and faecal parameters of altered intestinal permeability might be related to this new parameter.

## Data Availability

Data and material are available for anyone who concerns by request (email).

## Abbreviations

MD: Ménière disease
FL: functional level
vs: versus
